# EgoFall: Real-Time Privacy-Preserving Fall Risk Assessment With a Single On-Body Tracking Camera

**DOI:** 10.1109/TNSRE.2025.3577550

**Published:** 2025

**Authors:** Chiao-Yi Wang, Faranguisse Kakhi Sadrieh, Yi-Ting Shen, Giovanni Oppizzi, Li-Qun Zhang, Yang Tao

**Affiliations:** Department of Bioengineering, University of Maryland, College Park, MD 20742 USA; Department of Bioengineering, University of Maryland, College Park, MD 20742 USA; Department of Electrical and Computer Engineering, University of Maryland, College Park, MD 20742 USA.; Department of Bioengineering, University of Maryland, College Park, MD 20742 USA; Department of Bioengineering, University of Maryland, College Park, MD 20742 USA; Department of Bioengineering, University of Maryland, College Park, MD 20742 USA

**Keywords:** Fall risk assessment, body-worn tracking camera, ego-body motion, CNN-Transformer

## Abstract

Falls are a leading cause of injury among older adults, with research indicating that they often fall due to certain individual biomechanical factors. Therefore, real-time individual fall risk assessment is essential for designing more effective fall prevention programs and developing advanced home-based training solutions. However, existing methods for fall risk assessment either raise privacy concerns due to sensors installed in the environment or require multiple wearable devices, limiting their practicality for home-based applications and long-term monitoring. In this paper, we introduce EgoFall, a real-time privacy-preserving fall risk assessment system. EgoFall utilizes a chest-mounted commercial tracking camera and a carefully designed data pre-processing pipeline to acquire the ego-body motion data of the subject. The data is then fed to a lightweight CNN-Transformer model for fall risk assessment. To evaluate the proposed method, we establish the EgoWalk dataset, which includes four walking patterns: normal, anterior-posterior instability, medial-lateral instability, and combined instability. Experimental results show that EgoFall achieves an accuracy exceeding 95% on the EgoWalk dataset, outperforming baseline methods while maintaining low computational complexity. Additionally, a series of ablation studies explore the impact of fine-tuning data and error analysis, further highlighting EgoFall’s practicality in real-world applications.

## I. Introduction

Falls are the leading cause of injuries among elderly adults. Statistics show that approximately 30% of elderly adults in the US experience at least one fall each year, and the rate of fall-related injuries increases with age [1]. In 2020 alone, there were 3 million fall-related emergency room visits in the US, resulting in a total medical cost of $50 billion dollars [2], [3]. Beyond the economic burden, frequent falls among elderly adults can lead to reduced levels of activity, increased fear of falling, and a decrease in quality of life [4]. These concerns underscore the urgent need for effective fall prevention, detection, and rehabilitation methods. One foundational step in addressing these needs is conducting a fall risk assessment, which helps identify individuals at higher risk and guides targeted interventions to prevent falls before they occur.

Fall risk assessment, which involves identifying factors that increase an individual’s likelihood of falling, can be informed by understanding the biomechanical causes of instability during walking. For instance, instability in the anterior-posterior (A-P) and medial-lateral (M-L) directions has been identified as a major cause of slip-induced falls among elderly adults [5]. These direction-specific instability patterns closely reflects an individual’s ability to control their center of mass (COM) relative to their base of support (BOS)—a key factor in maintaining balance. When COM motion (i.e., position and velocity) exceeds the limits of stability in either direction, the individual becomes susceptible to losing balance [5], [6], [7], [8], [9]. By identifying such patterns, clinicians can uncover underlying deficits in balance control, offering a deeper understanding of fall risk beyond simple prediction. Furthermore, recent studies suggest that elderly adults often fall due to consistent, individual-specific reasons [10]. Therefore, continuous, real-time monitoring and assessment of fall risk can help physical therapists design more effective, personalized fall reduction training programs [10], [11], [12], [13]. Moreover, timely fall risk assessment can also support home-based interventions, empowering elderly adults to actively engage in fall reduction training outside clinical settings.

To effectively assess fall risk and inform personalized interventions, a variety of methods have been developed to analyze walking and falling patterns. These methods can be broadly categorized into binary and multi-class classification approaches. Binary classification methods primarily focus on distinguish walking and falling patterns between two states—such as fall versus no fall [14], [15], [16], [17], or normal versus abnormal gait [18], [19], a subject extensively studied for decades [20], [21], [22], [23]. For example, Lu et al. [14] proposed a hybrid model that combined 3D CNN and LSTM for fall detection, achieving superior performance. Similarly, Salimi et al. [15] and Wu et al. [16] utilized human pose images generated from RGB video frames to detect falls. Qi et al. [17] developed a multimodal data fusion method for classifying falls and non-falls, integrating time-series data from wearable sensors and visual data from cameras. Furthermore, Rana et al. [18] and Burduk et al. [19] focused on binary classification to differentiate between normal and abnormal gait patterns.

Despite their simplicity, binary classification methods often lack the granularity needed for personalized fall reduction training or rehabilitation tailored to specific impairments. To address this, multi-class classification methods have been explored, aiming to categorize different types of falls [24], [25], recognize gait cycles [26], [27], or classify various gait patterns [28], [29], [30], [31]. For instance, Imamura et al. [24] utilized a Doppler radar sensor to classify different types of falls, while Ma et al. [25] proposed a method for detecting fall direction using FMCW radar. Additionally, Sadeghzadehyazdi et al. [28] and Chen et al. [29] employed skeleton data recorded by Kinect for abnormal gait classifications.

Beyond classification, the complexity of different hardware setups is another critical consideration. Existing methods can generally be categorized into two types based on their sensor configurations. The first type places sensors, such as RGB cameras [14], [16], [28] or radar sensors [24], [32], in the subject’s surrounding environment. While these methods typically require fewer devices, they may raise privacy concerns if the subject can be identified from the captured data. Moreover, the need to set up third-person perspective sensors makes these methods more suitable for controlled environments, such as laboratories or hospitals. The second type of methods involves installing sensors, such as IMUs [30], [33], directly on the subject’s body. This setup mitigates privacy concerns and allows for more flexible use across various locations and times. However, it often requires multiple sensors to be mounted on different parts of the body, making it less practical for home-based applications or long-term monitoring, particularly for older adults.

**Fig. 1.**
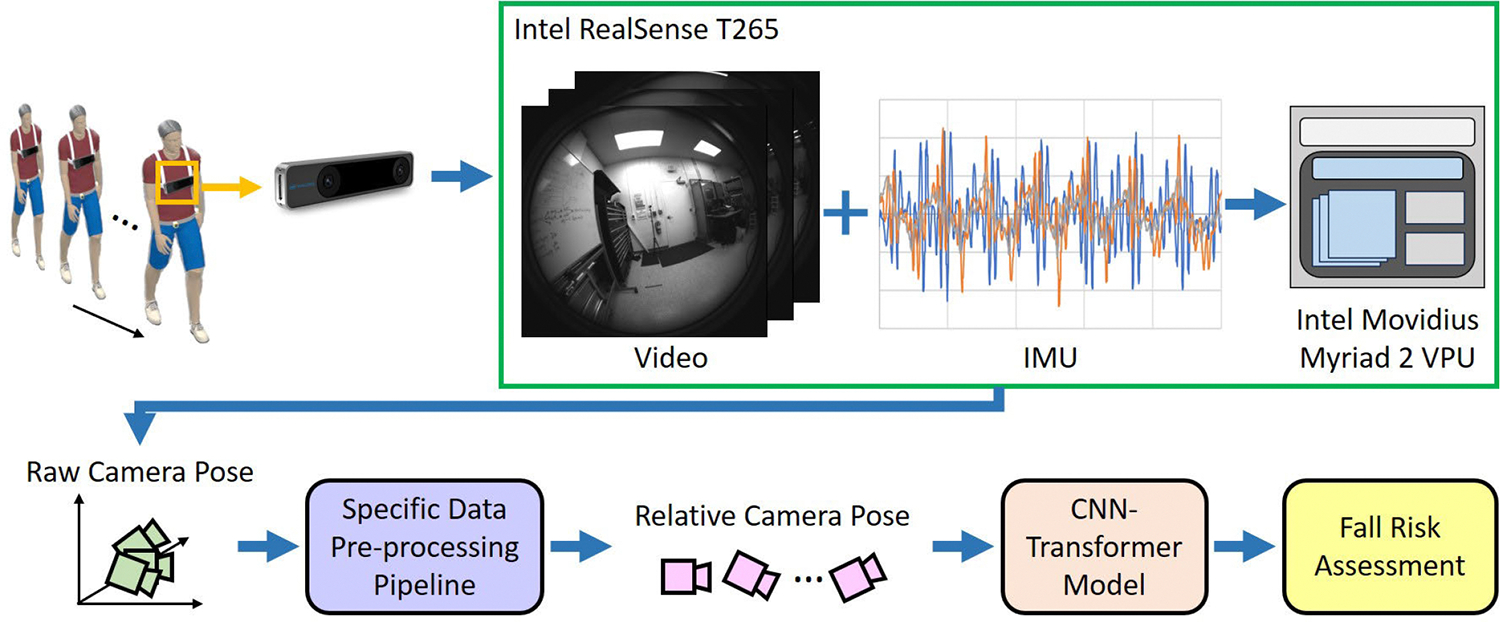
Overview of the proposed EgoFall System. An Intel RealSense^*™*^ camera is mounted on the subject’s chest to capture egocentric video frames and IMU data. These data are then processed internally by the vision processing unit (VPU) in the Intel RealSense camera to estimate the subject’s ego-motion in terms of relative camera poses. After pre-processing, the relative camera poses are fed into a lightweight CNN-Transformer model to classify the subject’s walking patterns.

To address the limitations mentioned above, we introduced EgoFall in our previous conference publication [34]. EgoFall is designed to identify direction-specific instability patterns, providing clinically meaningful information that can assist physical therapists in designing more targeted and personalized fall reduction programs though real-time fall risk assessment. An overview of the EgoFall system is presented in Fig. 1. EgoFall employs a chest-mounted tracking camera to capture ego-body motion (i.e., the camera poses) of the subject. With a carefully designed pre-processing pipeline and a lightweight CNN-Transformer model, EgoFall demonstrated superior performance compared to baseline methods on our EgoWalk dataset containing three types of walking patterns (i.e., normal, A-P instability, and M-L instability). Requiring only a single sensor, the EgoFall system is simple and holds significant potential for home-based fall reduction training.

This paper extends our previous conference work [34], with the goal of substantially enhancing its applicability in more practical, real-world scenarios. In particular, since real-world instabilities often involve a combination of A-P and M-L movements, we expanded our EgoWalk dataset to include a fall type that combines these directions. Additionally, we increased the dataset size by including more human subjects. Even though the classification difficulty significantly increased with the new dataset, we demonstrated that our CNN-Transformer model could still achieve high performance with minimal subject-specific fine-tuning data. Furthermore, we conducted a series of ablation studies, including an investigation into the impact of varying amounts of fine-tuning data and a detailed error analysis, to highlight the practicality of the EgoFall system. These studies were not included in our previous work [34]. In summary, the contributions of this paper are as follows:

- We significantly expanded the EgoWalk dataset [34] by adding an additional walking pattern (a combination of A-P and M-L directions) and increasing the number of subjects. The expanded dataset now comprises four walking patterns and 26 subjects (previously, three patterns and 15 subjects).
- We demonstrated that the proposed CNN-Transformer model achieves superior performance on the expanded dataset using minimal subject-specific fine-tuning data.
- We conducted additional ablation studies on the effect of fine-tuning data and error analysis to further validate the practicality of the EgoFall system in real-world scenarios.

The remainder of the paper is organized as follows: Section II introduces the EgoWalk dataset. Section III provides a detailed description of the proposed EgoFall system. Section IV outlines our experimental settings. Section V presents the results along with ablation studies, and Section VI provides a detailed performance and error analysis, including their correlation to the dataset characteristics. Finally, Section VII concludes the paper.

## II. The EgoWalk Dataset

### A. Background

Fall detection has been extensively studied for decades, leading to the availability of several publicly accessible datasets. These datasets can be broadly classified into three categories: vision-based, sensor-based, and multimodal.

Vision-based datasets, such as Le2i [35], FDD [36], the Multiple Cameras Fall Dataset [37], SDUFall [38], and EDF [39], consist of images captured using RGB cameras or Kinect devices. Sensor-based datasets, including FallAllD [40], SisFall [41], KFall [42], DLR [43], MobiFall [44], and UMAFall [45], contain data collected from inertial measurement units (IMUs), accelerometers, or gyroscopes. Multimodal fall datasets, which integrate both vision-based and sensor-based data, are relatively rare. For example, UR Fall [46] includes data from one IMU and two Kinects, while UP-Fall [47] combines data from five IMUs, one EEG, six infrared sensors, and two cameras. Although some of these datasets account for different falling directions and a variety of daily activities, none consider different unstable walking patterns—the primary focus of this work.

Beyond fall detection, some datasets focus on pathological gaits, such as the Walking Gait Dataset [48], the MMGS Dataset [49], the Pathological Gait Dataset [50], and the NDD Database [51], which aim to provide a more fine-grained analysis of walking patterns. However, these datasets primarily rely on multiple wearable sensors or third-person perspective cameras, which may be impractical for home-based training for elderly individuals and could raise privacy concerns.

To evaluate the proposed EgoFall system, we established the EgoWalk dataset using a single chest-mounted tracking camera that captures the subject’s ego-motion. This setup is lightweight, making it suitable for daily use while also enhancing subject privacy. The details of the dataset are presented in the following sections.

### B. Hardware Setup

**Fig. 2.**
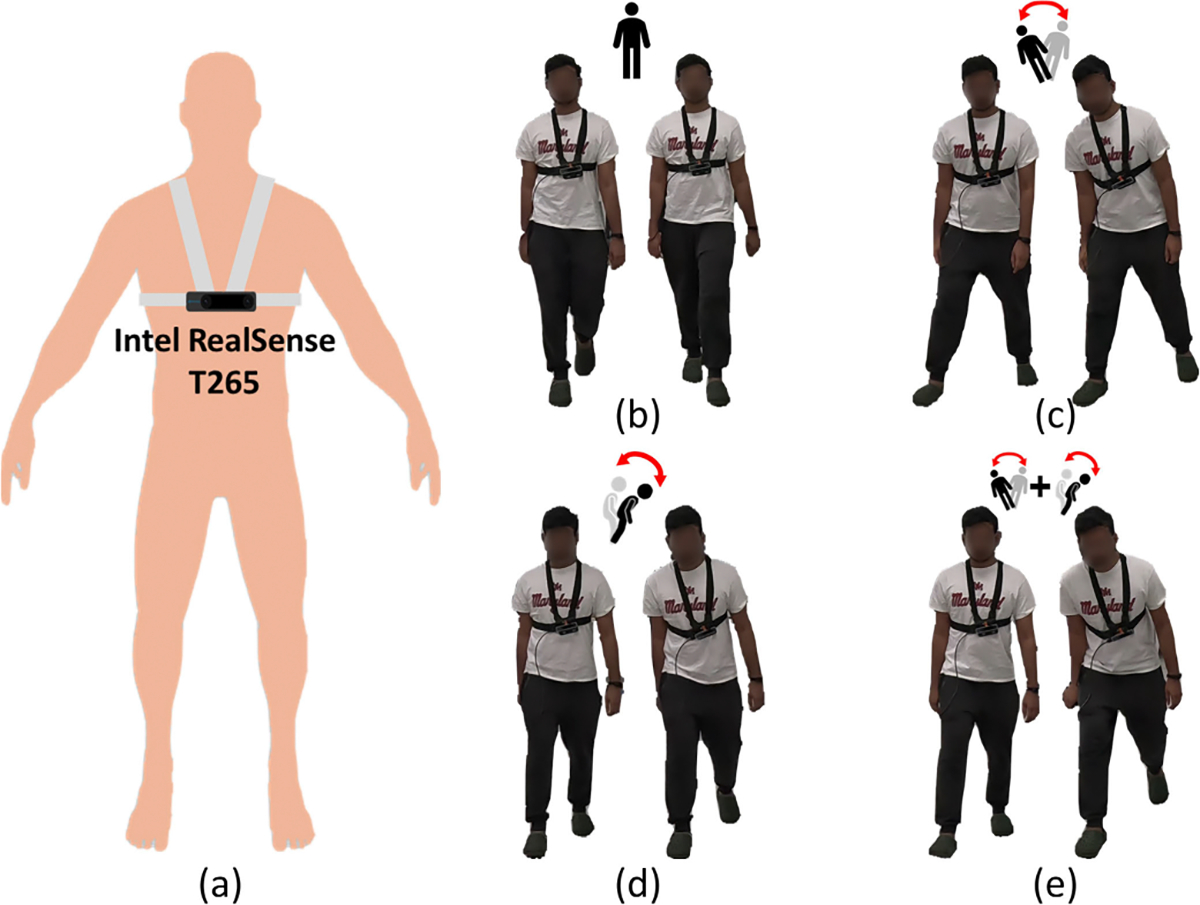
(a) Hardware setup of the EgoFall system and the considered four walking patterns: (b) normal, (c) medial-latera (M-L) instability, (d) anterior-posterior (A-P) instability, and (e) combined instability.

Our hardware setup is shown in Fig. 2 (a). An Intel RealSense T265 tracking camera was mounted on the subject’s chest using an adjustable chest strap. The tracking camera is 108×25×13 mm in size and weighs only 55 grams, so it won’t put too much burden on the subject. It includes two fisheye lens sensors, an IMU and an Intel Movidius Myriad 2 Visual Processing Unit (VPU). The VPU is able to execute the entire visual-inertial simultaneous localization and mapping (VI-SLAM) algorithm onboard in real-time [52] to provide its own 6 degrees of freedom (6-DoF) camera pose, including orientation and translation, in 3D space. The positive X, Y, and Z directions correspond to the right, top, and back of the tracking camera, respectively.

### C. Data Collection

We established a dataset called EgoWalk to validate the proposed EgoFall system. All data were collected following a protocol approved by the Institutional Review Board (IRB) of the University of Maryland, Baltimore (Protocol HP-00103991, approval date: May 04, 2023). EgoWalk consists of two subsets: EgoWalk-3 and EgoWalk-4, containing three and four different walking patterns, respectively. EgoWalk-3 includes normal walking, medial-lateral (M-L) instability, and anterior-posterior (A-P) instability. This subset was collected from 26 healthy volunteers of varying heights and weights, comprising 21 males and 5 females. EgoWalk-4 includes the same three walking patterns as EgoWalk-3, with an additional pattern that combines M-L and A-P instability (i.e., *combined* instability). This subset was collected from 16 healthy participants, including 13 males and 3 females.

**Fig. 3.**
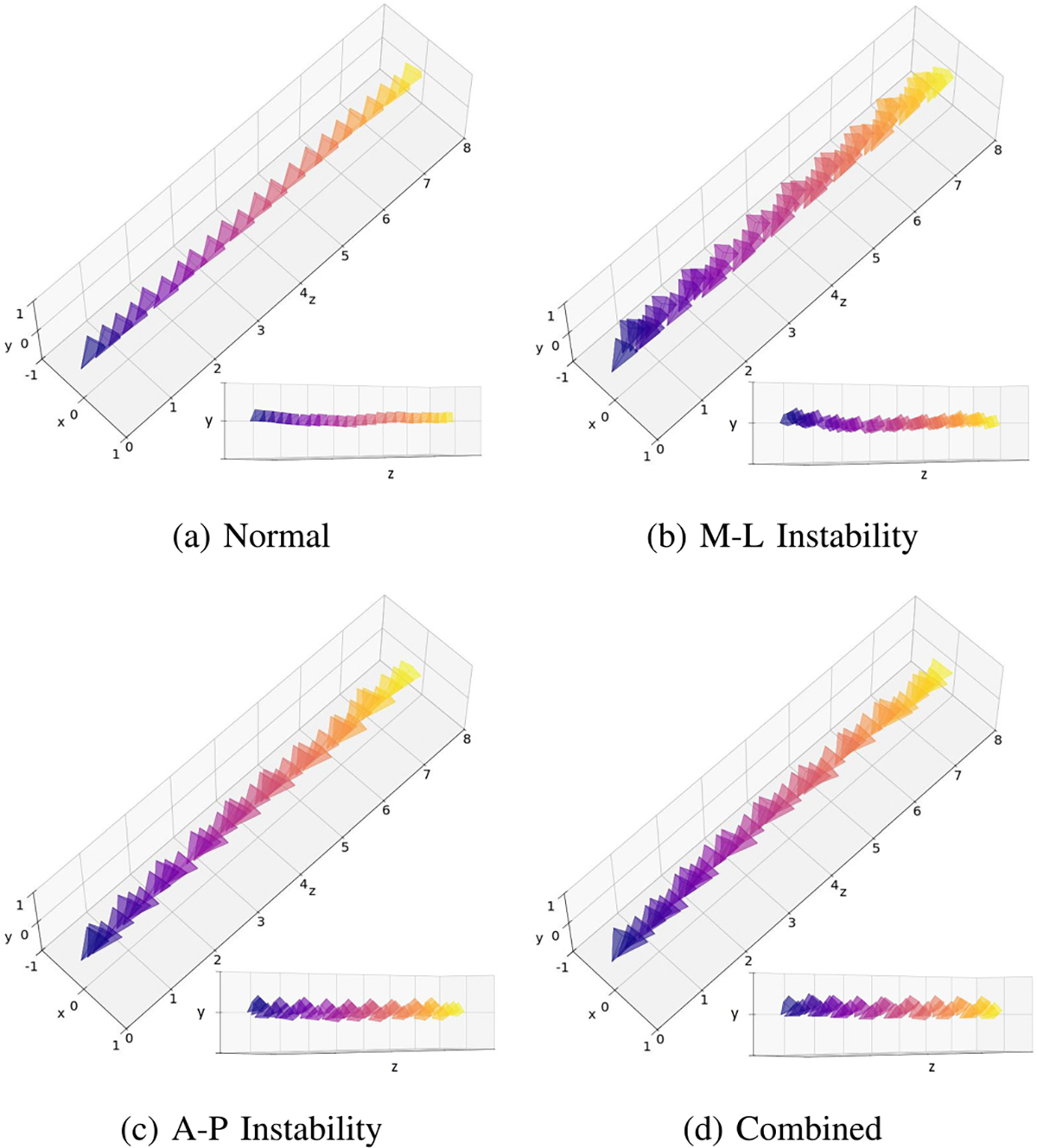
Examples of camera pose sequence for the four different walking patterns. Each sequence is shown from two different elevation angles (0° and 60°). M-L instability exhibits more horizontal movement, while A-P instability shows more vertical movement.

**Fig. 4.**
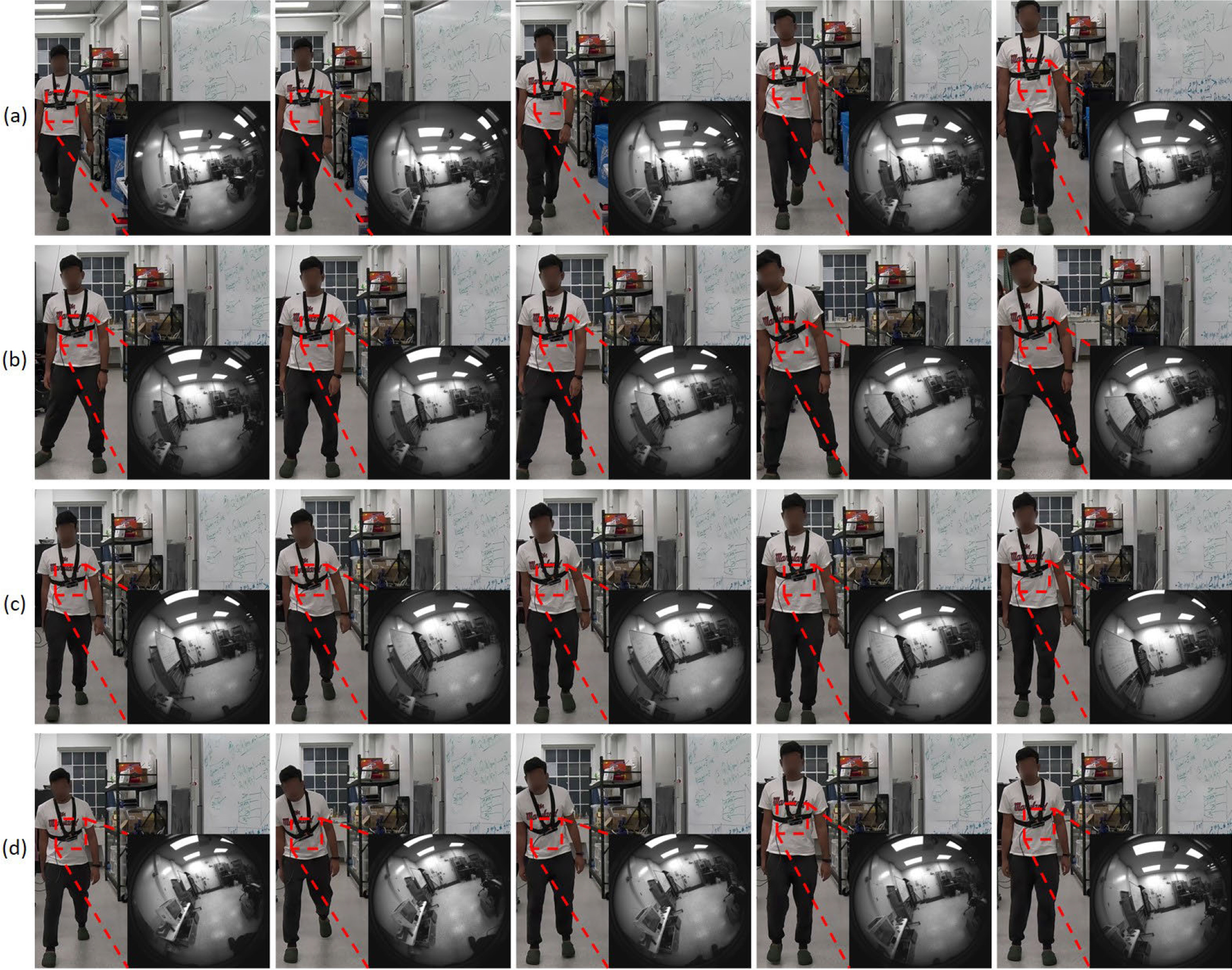
Examples of image sequences from both first-person and third-person perspectives for the four walking patterns: (a) normal, (b) M-L instability, (c) A-P instability, and (d) combined instability. The third-person perspective images are for illustration purposes only and are not included in the EgoFall system.

During data collection, each participant walked an 8-meter distance and performed each walking pattern twice. Based on well-defined biomechanical criteria for instability [5], participants were explicitly instructed to alter their center of mass (COM) trajectory during each step to reflect these instability types. Camera poses were extracted at a rate of 30 Hz. Examples of the camera pose sequences for the four different walking patterns are shown in Fig. 3. A summary of the two subsets is provided in Table I, while Fig. 2 (b)–(e) illustrates the four different walking patterns. Additionally, images sequences from both the first-person and third-person perspectives for each walking pattern are presented in Fig. 4.

## III. The EgoFall System

The EgoFall system consists for two major components: a data pre-processing module and a CNN-Transformer model. After being captured by the Intel RealSense tracking camera, the camera pose sequence undergoes pre-processing to ensure independence from the starting location. The pre-processed sequence is then fed into a lightweight CNN-Transformer model to classify the walking pattern. Details of these two components are provided below.

### A. Data Pre-Processing

The Intel RealSense T265 tracking camera continuously generates the camera pose relative to the initial camera pose. The $i^{\mathrm{th}}$ camera pose in a camera pose sequence can be represented as a 7-dimensional vector, which can be written as

(1) $$p_{i}^{\left\{0\right\}}=\left(t_{x},t_{y},t_{z},r_{x},r_{y},r_{z},r_{w}\right).$$

**Fig. 5.**
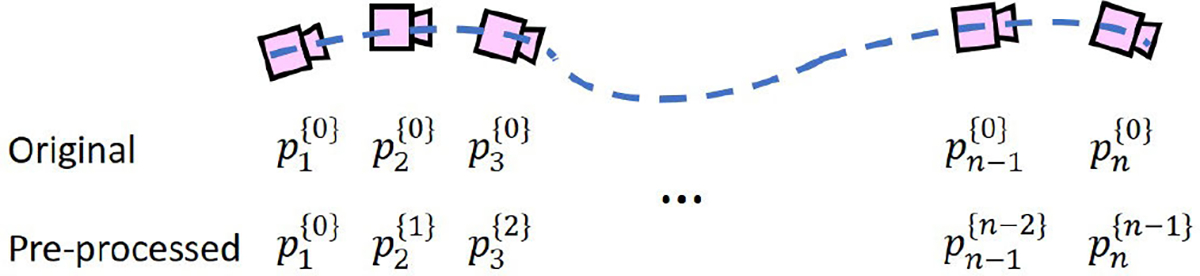
The original camera poses are relative to the initial camera pose, while our pre-processed camera poses are relative to their previous camera poses.

$p_{a}^{\left\{b\right\}}$ represents the $a^{\mathrm{th}}$ camera pose relative to the $b^{\mathrm{th}}$ camera pose. $t_{x}$, $t_{y}$, and $t_{z}$ represent the X, Y, and Z values of translation in meters. $r_{x}$, $r_{y}$, $r_{z}$, and $r_{w}$ correspond to the $Q_{i},\;Q_{j}$, $Q_{k}$, and $Q_{r}$ components in the quaternion representation of rotation. Since the initial camera pose differs in each walking trial, we found that such a representation of the camera pose was not optimal for a model to learn effective features for fall risk assessment. Therefore, instead of using the camera pose relative to the *initial* camera pose (i.e., $p_{i}^{\left\{0\right\}}$), we used the camera pose relative to the *previous* camera pose (i.e., $p_{i}^{\left\{i-1\right\}}$), as shown in Fig. 5. We calculated the new camera pose representation using the following equations:

(2) $$p_{i}^{\left\{0\right\}}\equiv P_{i}^{\left\{0\right\}}=\left[\begin{array}{cc} R_{i} & T_{i}\\ 0 & 1 \end{array}\right],$$

(3) $$p_{i}^{\left\{i-1\right\}}\equiv P_{i}^{\left\{i-1\right\}}=\left[\begin{array}{cc} R_{i-1}^{-1}R_{i} & R_{i-1}^{-1}\left(T_{i}-T_{i-1}\right)\\ 0 & 1 \end{array}\right].$$

$P_{a}^{\left\{b\right\}}$ is the 4 by 4 transformation matrix representing the $a^{th}$ camera pose relative to the $b^{\mathrm{th}}$ camera pose. $R_{a}$ and $T_{a}$ are the corresponding rotation and translation matrices. Note that $P_{a}^{\left\{b\right\}}$ and $p_{a}^{\left\{b\right\}}$ are two different representations of a relative camera pose and can be converted interchangeably. The pre-processing described above primarily involves basic matrix operations that are both computationally lightweight and memory-efficient, making them well-suited for real-time applications.

**Fig. 6.**
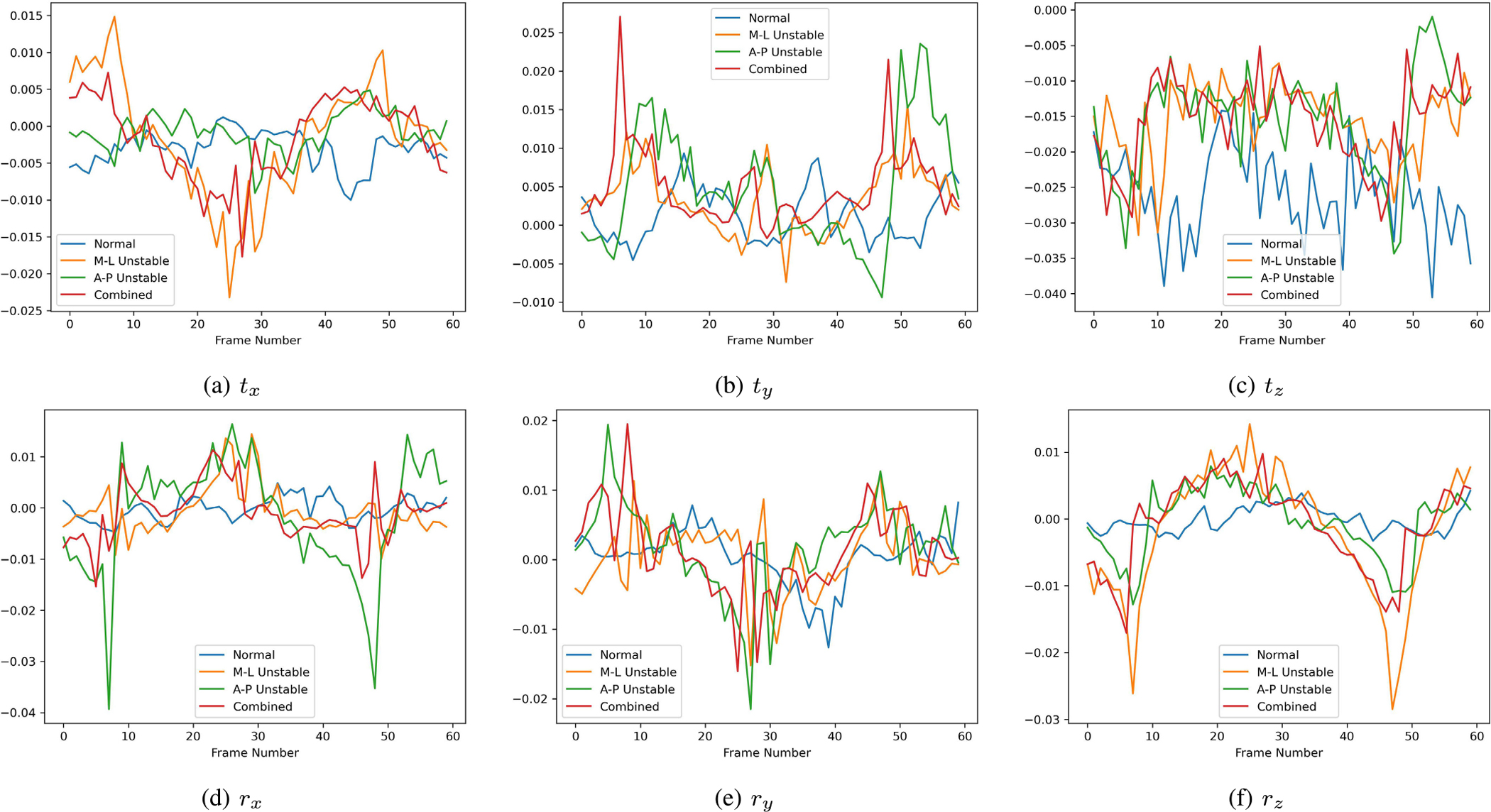
Six signals (after pre-processing) from four input tensors, each representing a different walking pattern. The y-axis units are in centimeters.

After data pre-processing, we concatenated every K consecutive camera poses into an input tensor of shape 7×1×K for our CNN-Transformer model. The value of K was selected to ensure that each input included at least one complete gait cycle. In this study, we chose K to be 60, which corresponds to a 2-second time window. To clarify, Fig. 6 presents six signals ($t_{x},t_{y},t_{z},r_{x},r_{y},r_{z}$) from four input tensors, each representing a different walking pattern. Although it is challenging to differentiate the walking patterns by merely observing the signals, some patterns are noticeable. Specifically, M-L instability tends to have more horizontal movements (Fig. 6a), while A-P instability is characterized by more vertical movements (Fig. 6b).

We also observed that the tracking camera occasionally generated incorrect tracking results. That is, a few of the collected camera pose sequences contain sudden and relatively large jumps in camera pose. In practice, this issue can be easily detected, allowing the system to issue a warning to the subject. In this study, we addressed this issue by introducing a maximum translation value, denoted as $t_{\max}$. Any relative camera pose with a translation value exceeding $t_{\max}$ was reset to static (i.e., no translation or rotation) throughout the experiments. $t_{\max}$ was set to be 0.1 meters based on the selected camera pose sampling rate and the typical human walking speed.

### B. Network Architecture

**Fig. 7.**
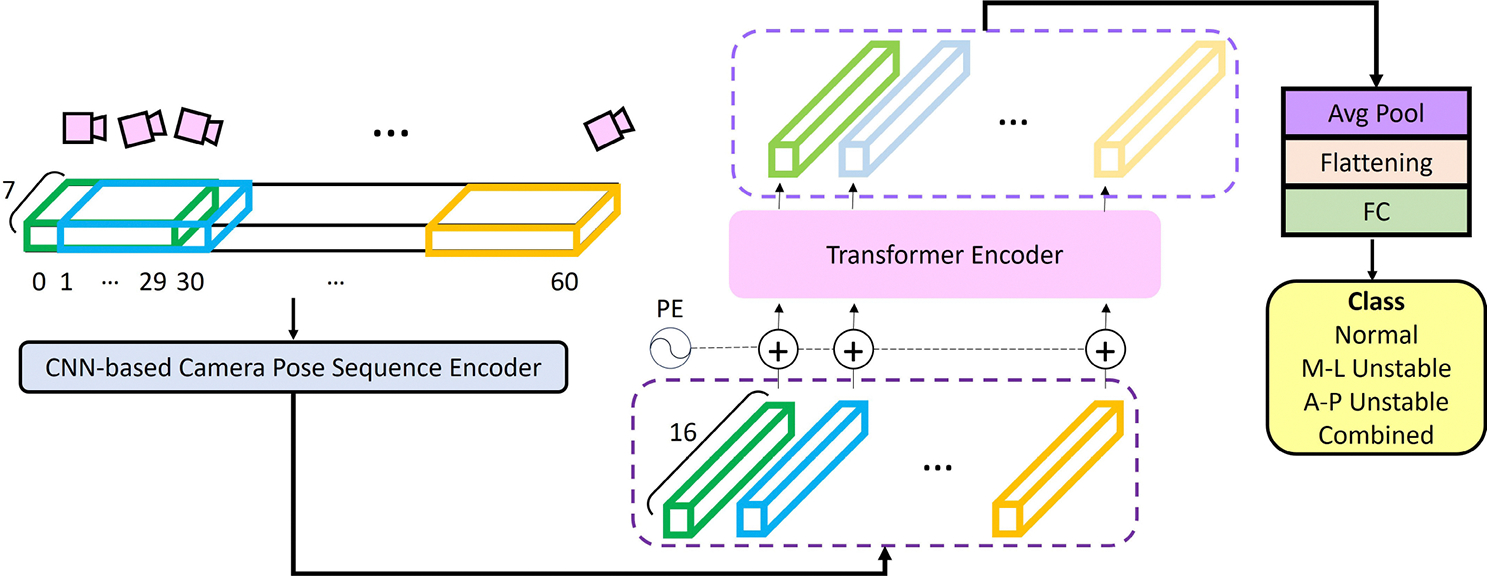
Overview of the proposed CNN-Transformer model. The input camera pose sequence, after pre-processing, is encoded by a CNN-based camera pose sequence encoder. After positional encoding, a transformer-based walking pattern predictor is employed to predict the subject’s walking pattern.

The proposed EgoFall system utilizes a lightweight CNN-Transformer model for real-time fall risk assessment. An overview of the model is shown in Fig. 7. It consists of two primary components: a CNN-based camera pose sequence encoder and a Transformer-based walking pattern predictor. First, the input tensor is processed by the camera pose sequence encoder. The encoder includes a 2-D convolution layer with D filters, a filter size of 1-by-N, and a stride of 1, followed by a ReLU layer. It encodes every N consecutive camera poses from the input tensor into a D-dimensional token, resulting in a total of (60-N+1) tokens. In this study, we selected N to be 30, which corresponds to a 1-second time window, and set D to be 16. That is, a total of 31 tokens were produced by our camera pose sequence encoder. Next, position embeddings [53] are added to the generated tokens to preserve positional information. The results are then fed into our Transformer-based walking pattern predictor. The predictor is composed of two Transformer encoder layers [53], followed by a linear layer. Each Transformer encoder layer contains a two-head self-attention layer and a feedforward network. In this study, a fixed latent dimension of 16 was adopted across the Transformer encoder layers. Finally, the output of the second Transformer encoder layer is averaged and used by the subsequent linear layer for the final prediction.

In summary, the proposed CNN-Transformer network encodes each short-term camera pose sequence with the CNN-based encoder and extracts long-term features among all short sequences with the Transformer-based predictor for fall risk assessment. In the next section, we will demonstrate that combining CNN and Transformer achieved better results than using either of them alone on our collected dataset.

### C. Subject-Specific Fine-Tuning

Although our CNN-Transformer model performs well for three walking patterns (EgoWalk-3), we observed a decrease in accuracy when it is directly applied to four walking patterns (EgoWalk-4). This decline can be attributed to two primary factors: 1) The *combined* walking pattern is inherently more difficult to distinguish from the A-P and M-L patterns, as it incorporates characteristics of both instabilities. 2) Walking patterns vary among individuals, even when performing the same walking patterns, which adds to the classification complexity. Despite these challenges, we demonstrate that our CNN-Transformer model can regain its high performance by fine-tuning with a minimal amount of subject-specific data (e.g., 16 seconds) in Sec. V-B.

## IV. Experimental Setups

### A. Dataset

We conducted experiments using both the EgoWalk-3 and EgoWalk-4 subsets (Tab. I). The EgoWalk-3 subset comprises 52 camera pose sequences for each walking pattern from 26 subjects, while the EgoWalk-4 subset includes 32 sequences for each walking pattern from 16 subjects. Each subject was asked to perform the same walking pattern twice. For the EgoWalk-3 subset, 16 subjects were assigned to the training set, 5 to the test set, and the remaining 5 to the validation set. Similarly, for the EgaoWalk-4 subset, 10 subjects were assigned to the training set, 3 to the test set, and the remaining 3 to the validation set. To perform subject-specific fine-tuning III-C, one camera pose sequence of each walking pattern from the subjects in the test set was used for fine-tuning, while the other was used for testing. A summary of the dataset splits and the statistics for each subset after the data pre-processing steps described in Sec. III-A are presented in Table II.

### B. Baseline Methods

We compared our CNN-Transformer model with five baseline methods that are representative and widely applied in fall detection research. Specifically, we selected Support Vector Machine (SVM), a well-known machine learning method for data classification, which was employed by Rana et al. [18] and Burduk et al. [19] to recognize gait abnormalities. Among deep learning-based methods, CNN [15], [29], [30], LSTM [24], [26], [29], and CNN-LSTM [15], [28] have been applied to tasks such as multi-class fall detection, gait classification, gait recognition, and gait phase estimation, demonstrating strong performance in these areas. For a fair comparison, we maintained a similar scale of network parameters across all deep learning-based methods (as shown in Tab. III). The implementation details of these five baseline methods are as follows:

- **Support Vector Machine (SVM)**: We implemented SVM using the sklearn library with the default settings. The input tensors were flatten before being used in this case.
- **Convolutional Neural Network (CNN)**: We attached a 2-D max pooling layer with a kernel size of 1 × 2 to our CNN-based camera pose sequence encoder. The output was then passed through another 2-D convolution layer with 16 filters, a filter size of 1 × 8, and a stride of 1, followed by a ReLU layer and a global average pooling layer. A linear layer was used at the end for the final prediction.
- **Transformer**: We replaced our CNN-based camera pose sequence encoder with a linear layer. Everything else remained the same as our CNN-Transformer model.
- **Long Short-term Memory (LSTM)**: We implemented a vanilla LSTM with 2 layers and a hidden size of 16, followed by a linear layer for the final prediction.
- **CNN-LSTM**: We substituted our Transformer-based walking pattern predictor with the aforementioned LSTM-based predictor.

### C. Training, Fine-Tuning, and Testing Details

All models in this study were implemented using PyTorch. Training was conducted for 1600 epochs with a batch size of 256. The Adam optimizer was used to minimize the cross-entropy loss. The learning rate was initialized at 0.0002 and decayed by 20% every 400 epochs. The model achieving the highest accuracy on the validation set was selected for evaluation. During fine-tuning on the EgoWalk-4 subset (Sec. III-C), the model was fine-tuned for 50 epochs with a reduced learning rate of 0.0001. The model from the final epoch was selected for evaluation. Each experiment was repeated three times, and the average of all evaluation metrics reported. All training processes were executed on an NVIDIA GeForce RTX 3060 Ti GPU, while evaluations were conducted on an Intel i9–10900X CPU with a batch size of 1.

### D. Evaluation Metrics

Similar to our previous work [34], we followed [16] and used the following four evaluation metrics:

(4) $$\mathrm{Accuracy}=\frac{TP\;+\;TN}{TP\;+\;TN\;+\;FP\;+\;FN},$$

(5) $$\mathrm{Recall}\;(R)=\frac{TP}{TP\;+\;FN},$$

(6) $$\mathrm{Precision}\;(P)=\frac{TP}{TP\;+\;FP},$$

(7) $$F1\;\mathrm{score}=\frac{2\;\times \;P\;\times \;R}{P\;+\;R}.$$

TP,TN,FP, and FN represent true positives, true negatives, false positives, and false negatives, respectively. The Recall,Precision, and F1score reported are the average across all classes.

## V. Results

### A. Results on the EgoWalk-3 Subset

Table IV presents the results of our method compared to the baseline methods on the EgoWalk-3 subset. Overall, our method outperformed all the baseline methods while maintaining comparable computational complexity and real-time performance (as shown in Tab. III). We also observed that SVM performed the worst, possibly due to the high noise levels in the collected ego-body motion data, which made it difficult for SVM to learn effectively. The other four baseline methods generally performed well, but their accuracy was still 3% to 7% lower than that of our CNN-Transformer model. This highlights the advantages of combining CNN and Transformer.

### B. Results on the EgoWalk-4 Subset

Table V presents the results of our method and the baseline methods on the EgoWalk-4 subset. The subject-specific fine-tuning results (Tab. Vb) represent the average performance across three different subjects, each fine-tuned individually. During subject-specific fine-tuning, all baseline methods were fine-tuned using the same data as our method, as described in Sec. III-C, with all other setting kept as default.

From Table V, it is evident that all methods performed worse without subject-specific fine-tuning compared to their performance on the EgoWalk-3 subset (Tab. IV). As discussed in Sec. III-C, one possible reason for this is that the *combined* walking pattern exhibits characteristics of both A-P and M-L instabilities, making it more challenging to distinguish. Additionally, individual differences in each walking pattern contribute significantly to the decline in accuracy.

After applying subject-specific fine-tuning, the accuracy of all methods improved significantly, ranging from 15% to 26%. Notably, our CNN-Transformer model outperformed all baseline methods by a large margin across all evaluation metrics, achieving an accuracy of 95%. This demonstrates that our CNN-Transformer is more effective at differentiating the *combined* walking pattern from the other walking patterns. We attribute this to the complementary strengths of both CNN and Transformer components. The CNN-based camera pose sequence encoder effectively extracts features of different walking patterns from continuous 30-frame (i.e., 1-second) camera pose data. Meanwhile, the Transformer-based walking pattern predictor excels at capturing long-term dependencies across every token (i.e., the extracted feature from every 30-frame camera pose data).

### C. Ablation Study: Data Pre-Processing

In this section, we demonstrate the benefits of the new camera pose representation (i.e., the camera pose after our pre-processing step) described in Sec. III-A. To evaluate its effectiveness, we also trained our CNN-Transformer model using the original camera pose representation, keeping all other settings unchanged. We conducted the experiment on both the EgoWalk-3 and EgoWalk-4 subsets, with the results presented in Table VIa and Table VIb. Compared to training on the pre-processed camera poses, training on the original camera poses significantly reduced performance on both datasets. As discussed in Sec. III-A, the original camera pose representation defines each camera pose relative to the initial camera pose. However, since the initial camera pose varies across trials, models trained on the original camera pose representation exhibited poor generalization to the test set. In contrast, training models on the pre-processed camera poses effectively resolved this issue, leading to improved accuracy and robustness.

### D. Ablation Study: Subject-Specific Fine-Tuning

For subject-specific fine-tuning on the EgoWalk-4 subset, one camera pose sequence of each walking pattern from the subjects in the test set was used. These sequences covered the entire duration of the activity, with the maximum frame number for a single pose sequence being 480 frames (i.e., 16 seconds, as all sequences were recorded at 30 frames per second). In this section, we investigated whether using shorter camera pose sequences would affect performance and aimed to determine the minimum sequence length required for subject-specific fine-tuning to achieve satisfactory results. Specifically, we fine-tuned our CNN-Transformer model using first 15, 10, 8, and 5-second camera pose sequences from the complete sequences previously used in the fine-tuning set. All other settings were kept as default.

The results are shown in Table VII. As expected, we found that using longer camera pose sequences during subject-specific fine-tuning resulted in better performance. For example, accuracy decreased by 11% when a maximum sequence length of 150 (i.e., 5 seconds) was used for fine-tuning. However, performance remained satisfactory when using a maximum sequence length of 240 (i.e., 8 seconds) for fine-tuning, achieving an accuracy of over 90%. This finding highlights two key insights. First, five seconds of camera pose sequence per walking pattern may not provide sufficient information for effective fine-tuning, as it typically contains fewer than three walking cycles or fewer than five steps when a person is walking at a normal speed. A walking cycle is defined as the complete movement of both feet in one sequence. Second, this result demonstrates the feasibility of our method for real-world applications. Since subject-specific fine-tuning requires as few as 8 seconds of camera pose sequence per walking pattern from a subject, the proposed method can be easily applied to new subjects by collecting a small amount of data per walking pattern.

## VI. Discussions

### A. Performance and Error Analysis

#### 1) Per-Walking Pattern:

**Fig. 8.**
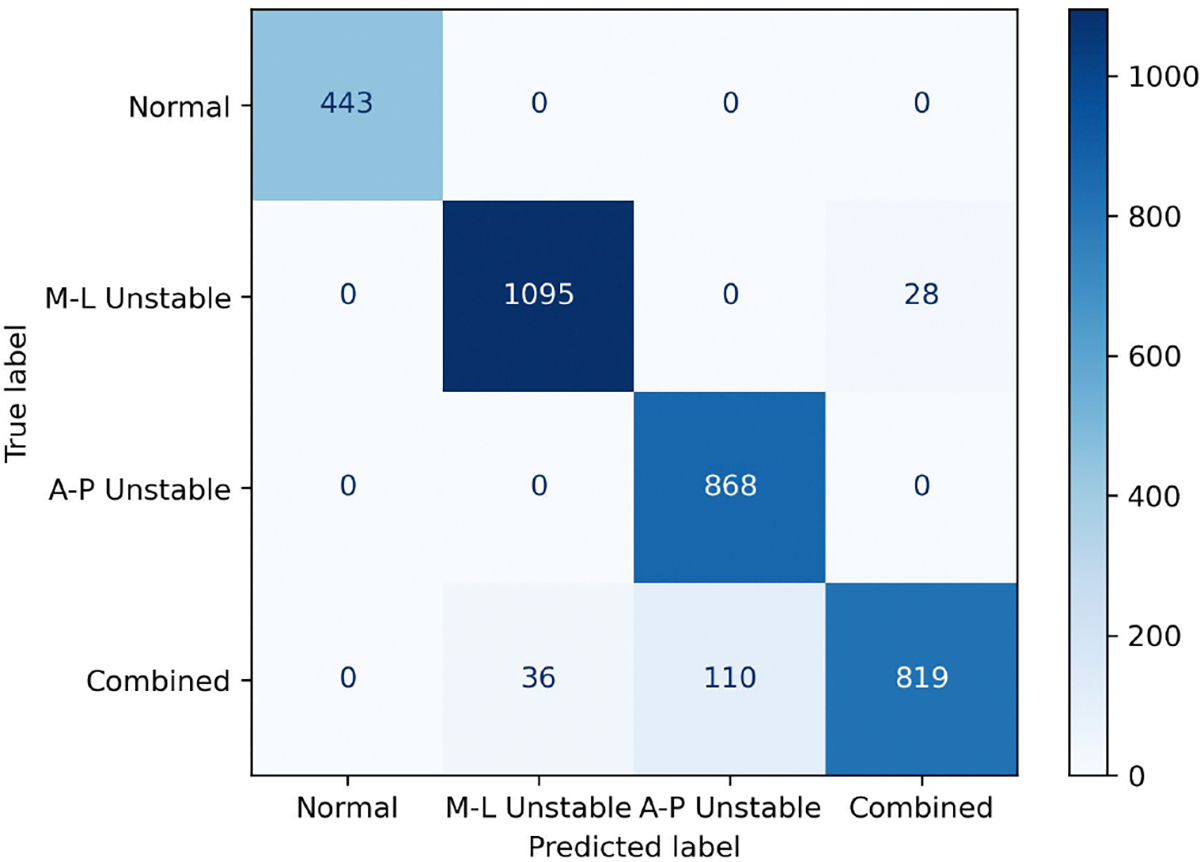
Confusion matrix of prediction results for different walking patterns.

Table VIII presents the average performance of our method for different walking patterns on the EgoWalk-4 subset, calculated over three experiments. Fig. 8 shows the confusion matrix of the prediction results for the four walking patterns, derived from one of these experiments. Among the four walking patterns, the *normal* walking pattern achieves the highest performance, reaching 1.0 across all four evaluation metrics. This indicates that the *normal* walking pattern exhibits the most distinct characteristics, making it easier to classify accurately. In contrast, the *combined* walking pattern shows the lowest accuracy. One possible reason is that the *combined* walking pattern exhibits a mix of characteristics from both M-L unstable and A-P unstable walking patterns, making it more challenging to classify. For example, Fig. 8 reveals that 11% of the *combined* walking pattern was misclassified as A-P instability, and 3% was misclassified as M-L instability. This suggests that the overlapping characteristics between these walking patterns contribute to the observed misclassifications.

#### 2) Per-Subject:

We examined the performance across three subjects in the test set of the EgoWalk-4 subset. Table IX shows the prediction accuracy for different walking patterns across subjects, calculated over three experiments. We found that the *combined* walking pattern consistently had the lowest accuracy across all subjects compared to other walking patterns, which aligns with the findings from the per-walking pattern analysis. However, the prediction accuracy for the *combined* walking pattern varied significantly across subjects, with some subjects achieving an accuracy of up to 0.98, while others only reached 0.67.

**Fig. 9.**
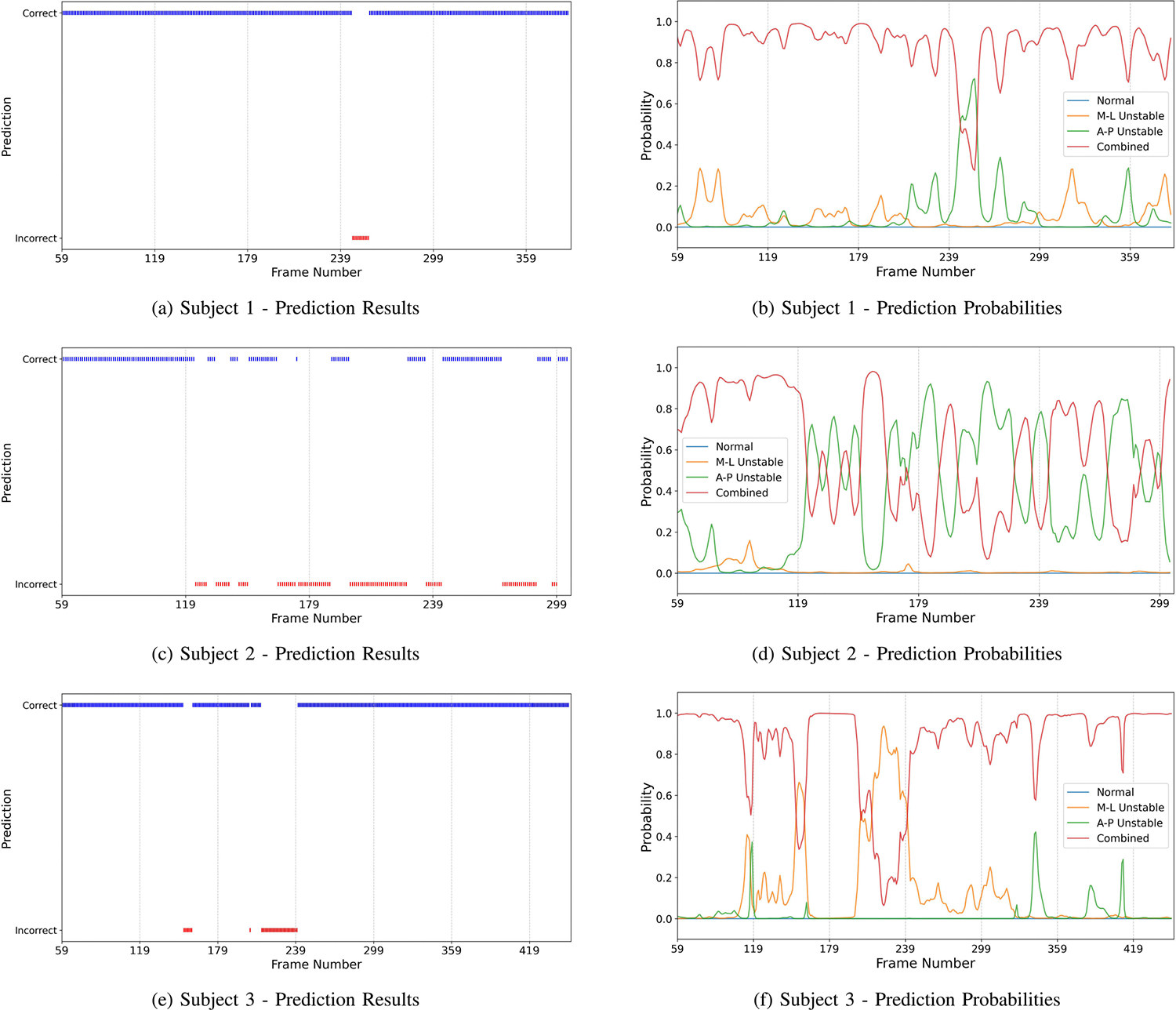
Predictions (a, c, e) and corresponding probabilities (b, d, f) generated by our model for the *combined* walking pattern of three subjects in the EgoWalk-4 test set. In the prediction figures (a, c, e), blue denotes correct predictions, while red indicates incorrect ones.

To gain further insights, we analyzed the predictions for each frame in the entire camera pose sequence, as shown in Fig. 9. We found that no frames were misclassified as the normal walking pattern. Additionally, some frames were misclassified as M-L instability, while others were misclassified as A-P instability. For the same subject, the misclassifications consistently corresponded to the same walking pattern, indicating that the missclassifications are influenced by each subject’s walking habits. For example, some subject’s *combined* walking pattern is more similar to M-L instability, while others resemble A-P instability. These findings suggest that individual walking patterns significantly influence the model’s prediction performance, highlighting the importance of subject-specific fine-tuning to enhance accuracy.

Moreover, we observed that misclassifications occurred periodically rather than in randomly separated frames. One possible explanation is that human walking is inherently periodic, and when subjects mimic the *combined* walking pattern, certain steps resemble either the A-P or M-L instabilities. As shown in the right column of Fig. 9, the prediction probabilities for some misclassifications are relatively low, ranging from 0.35 to 0.5, suggesting that these misclassifications could potentially be false alerts within the entire video. To address this issue and improve prediction accuracy, one potential solution is to set a threshold for the prediction probability or define the minimum number of consecutive frames classified as a certain walking pattern before the system confirms the prediction of a subject’s walking pattern. For example, if the prediction probability for a camera pose sequence is below the predefined threshold or if the number of consecutive frames classified as a walking pattern is insufficient, then it can be classified as a false alert. This approach enhances the model’s robustness by filtering out low-confidence predictions and preventing short-term misclassifications from affecting overall accuracy.

### B. Dataset Visualization

**Fig. 10.**
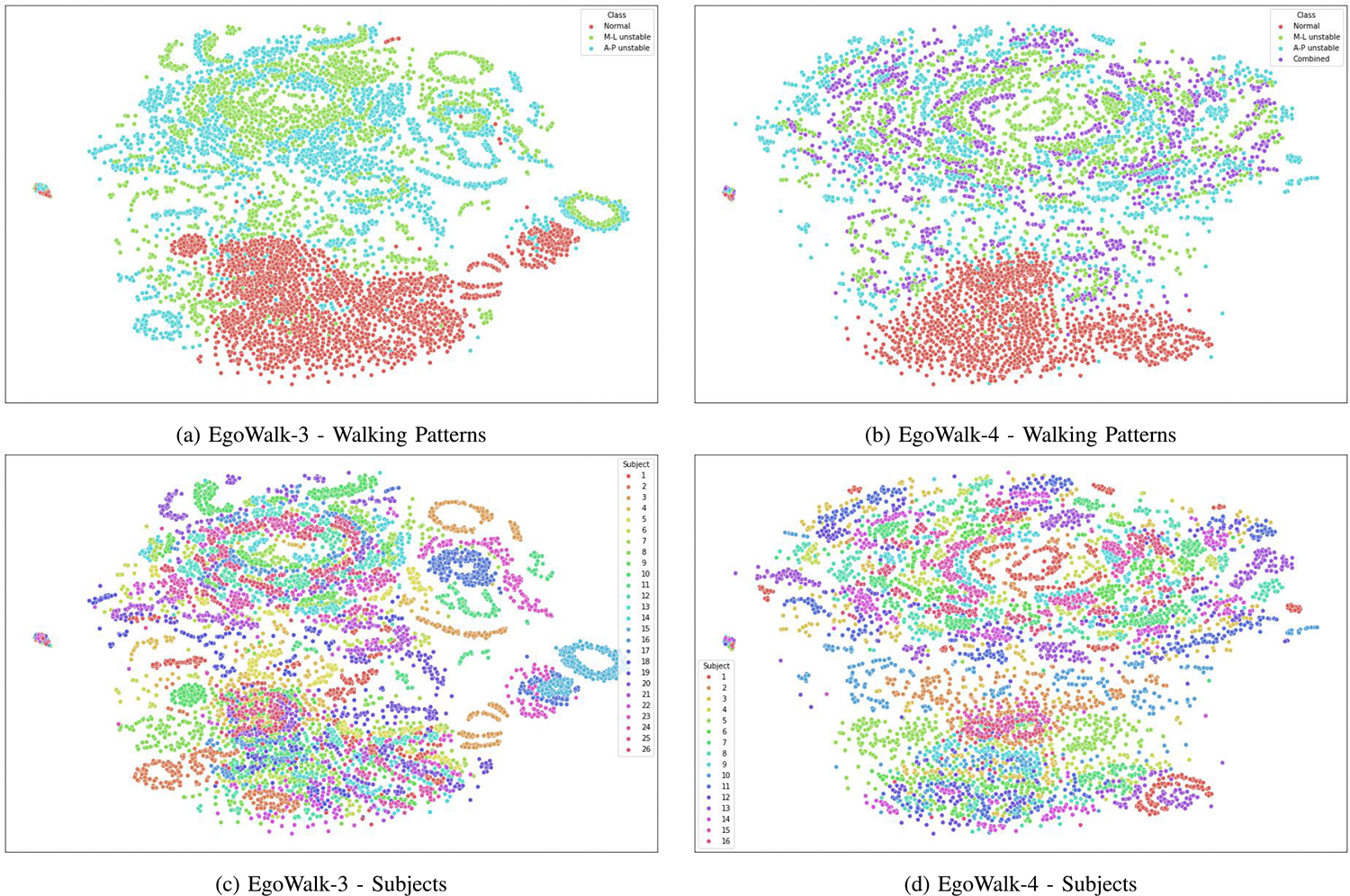
t-SNE visualization of the EgoWalk-3 and EgoWalk-4 subset. Each color represents either a different walking pattern ((a) and (b)) or a different subject ((c) and (d)). For optimal interpretation, please view in color.

To better understand the problem we are addressing, we applied t-SNE [54] to visualize EgoWalk-3 and EgoWalk-4 subsets after the data pre-processing step described in Sec. III-A (resulting in 7 × 1 × *K*-dimensional vectors) in a 2-dimensional space, as shown in Fig. 10. t-SNE maps data point similarities to joint probabilities and minimizes the Kullback-Leibler (KL) divergence between the joint probabilities of the low- and high-dimentional data. We used the *sklearn.manifold.TSNE* function with a perplexity of 100 and a maximum iteration of 5000. For each camera pose sequence, 50 data points were randomly sampled for visualization.

From Fig. 10, we observed that the *normal* walking pattern exhibits the most distinct characteristics compared to the other three walking patterns, consistent with our model’s superior performance on the normal walking pattern (Sec. VI-A.1). In contrast, the other three walking patterns tend to overlap, particularly in the EgoWalk-4 subset, explaining why most methods (including ours) struggle to distinguish them. Additionally, inter-subject differences are prominent, as one subject’s walking pattern may resemble another subject’s different walking patterns. This underscores the importance of the subject-specific fine-tuning step. Finally, the ring-like patterns observed in the t-SNE figures may reflect the periodic nature of the camera pose sequences data.

### C. Real-World Applicability and System Flexibility

EgoFall has low computational requirements and achieves real-time performance, making it practical for everyday use (Table III). It employs the compact Intel RealSense T265 camera—measuring 108 mm × 24.5 mm × 12.5 mm, weighing 55 grams, and consuming only 1.5 watts—while providing highly accurate camera pose data. The single chest-mounted camera eliminates the need for multiple body-worn sensors (e.g., IMUs), making the system more user-friendly, especially for older adults or non-technical users.

Importantly, EgoFall is not restricted to the T265. Because it relies solely on camera pose data derived from visual-inertial odometry (VIO), it is compatible with any device capable of running a VIO algorithm. This includes many off-the-shelf consumer electronics—such as smartphones—that integrate a camera, IMU, and computing capabilities [55], [56], [57], and are typically designed for full-day operation. This hardware flexibility broadens EgoFall’s applicability to both continuous at-home monitoring and in-clinic assessments.

### D. Limitations and Future Work

#### 1) Toward Real-World Gait Patterns:

Collecting real-world data from patients presents significant ethical and logistical challenges. Consequently, the use of simulated data for initial method development and benchmarking is common and widely accepted in recent literature [28], [29]. For example, Sadeghzadehyazdi et al. [28] evaluated their method using three datasets—the Walking Gait Dataset [58], the MMGS dataset [49] and the Pathological Gait Dataset [50]—all based on healthy subjects simulating gait abnormalities. Our EgoWalk dataset, which includes 26 subjects and is constructed based on well-defined biomechanical criteria for instability, follows a similar methodology and is comparable in scope.

However, we acknowledge that the simulated gaits in the EgoWalk dataset, performed by instructed subjects, may not fully capture the subtlety and complexity of real-world pathological gait patterns. Therefore, we consider the use of simulated gaits a valuable and controlled starting point for instability pattern recognition. This approach provides a practical and meaningful foundation for the development and evaluation of our method. At the same time, we plan to collect data from real patients with clinically verified unstable gait patterns to enable more robust evaluations and enhance the clinical applicability of our system.

#### 2) Enhancing Privacy Preservation:

We acknowledge that the chest-mounted camera used in EgoFall may still capture some visual information from the surroundings. However, EgoFall does not use raw image data for gait pattern classification. Instead, it extracts camera pose data using an on-device visual-inertial simultaneous localization and mapping (VI-SLAM) algorithm. Unlike most existing camera-based approaches that rely on third-person perspective cameras installed in the environment—often capturing identifiable facial features and other sensitive visual data—EgoFall is designed around a wearable, chest-mounted camera that intentionally avoids capturing the user’s face. Importantly, once the camera poses are extracted, the original images are no longer needed and can be immediately deleted, significantly mitigating privacy risks.

Furthermore, recent work [59], [60] have demonstrated that camera pose estimation can also be achieved using only inertial data through inertial odometry. We aim to integrate such approaches into future versions of EgoFall to further enhance privacy by eliminating the need to process any visual data.

## VII. Conclusion

In this paper, we introduced EgoFall, a real-time, privacy-preserving fall risk assessment system that leverages a chest-mounted commercial tracking camera to capture the subject’s ego-body motion. EgoFall features a simple hardware setup and integrates a carefully designed data pre-processing pipeline with a lightweight CNN-Transformer model, ensuring efficient and accurate performance. To demonstrate its applicability in real-world scenarios, we curated the EgoWalk dataset, which includes four walking patterns and a diverse ground of subjects. Comprehensive experiments demonstrated that the proposed CNN-Transformer model significantly outperforms baseline methods on the EgoWalk dataset. Furthermore, we conducted ablation studies to evaluate the impact of fine-tuning data and performed detailed error analyses, validating the robustness and practicality of the EgoFall system in real-world scenarios.

For future work, we plan to compare the performance of our method with approaches that utilize third-person perspective systems. Furthermore, to explore EgoFall’s potential for home-based fall reduction training, we intend to collect data from patients and apply our method to patient-specific scenarios. We hope that EgoFall will provide valuable insights for physical therapy and contribute to advanced home-based fall prevention training for older adults.

## Figures and Tables

**TABLE I:** Summary of the EgoWalk-3 and EgoWalk-4 Subsets

Subset	Subject	Train/Val/Test	Walking Pattern	Sequence

EgoWalk-3	26	16/5/5	Normal	52
			M-L Instability	52
			A-P Instability	52

EgoWalk-4	16	10/3/3	Normal	32
			M-L Instability	32
			A-P Instability	32
			Combined	32

**TABLE II:** Statistics for the Training, Validation, and Test Splits of the EgoWalk-3 and EgoWalk-4 Subsets. Each Number Represents the Data Volume (i.e., *K* Consecutive Camera Poses) After the Data Pre-Processing Steps Described in Sec. III-A

Subset	Walking Pattern	Train	Validation	Test	

				Fine-tune	Test

EgoWalk-3	Normal	6049	2164	907	863
	M-L Instability	11551	4817	1861	1889
	A-P Instability	10906	3567	1441	1399

EgoWalk-4	Normal	4045	896	503	443
	M-L Instability	7931	2246	1118	1123
	A-P Instability	7006	1741	875	868
	Combined	6334	1926	968	965

**TABLE III:** Number of Model Parameters and Runtime for Different Methods

Method	Parameters (k)	Runtime (ms)

LSTM	3.8	2
CNN-LSTM	7.8	1.25
Transformer	3.6	0.63
CNN	5.5	0.15
CNN-Transformer (Ours)	6.8	0.69

**TABLE IV:** Results of Different Methods on the EgoWalk-3 Subset. The Best Results Are Marked in **Bold**

Method	Accuracy	Precision	Recall	F1

SVM	0.61	0.42	0.62	0.50
LSTM	0.94	0.94	0.95	0.94
CNN-LSTM	0.90	0.89	0.91	0.90
Transformer	0.93	0.92	0.94	0.93
CNN	0.93	0.92	0.94	0.93
CNN-Transformer (Ours)	**0.97**	**0.96**	**0.97**	**0.96**

**TABLE V:** Results of Different Methods (a) With and (b) Without Subject-Specific Fine-Tuning on the EgoWalk-4 Subset. The Best Results Are Marked in **Bold**

	
Method	Accuracy	Precision	Recall	F1	Method	Accuracy	Precision	Recall	F1
	
SVM	0.43	0.34	0.44	0.35	SVM	-	-	-	-
LSTM	0.66	0.73	0.72	0.70	LSTM	0.82	0.85	0.83	0.84
CNN-LSTM	0.62	0.72	0.67	0.67	CNN-LSTM	0.87	0.89	0.89	0.88
Transformer	0.64	0.68	0.70	0.68	Transformer	0.84	0.86	0.86	0.85
CNN	**0.71**	0.76	**0.76**	**0.75**	CNN	0.86	0.88	0.88	0.88
CNN-Transformer (Ours)	0.69	**0.77**	0.75	0.73	CNN-Transformer (Ours)	**0.95**	**0.96**	**0.96**	**0.96**
	
(a) Without Subject-Specific Fine-tuning					(b) With Subject-Specific Fine-tuning				

**TABLE VI:** Comparison of Raw and Pre-Processed Data on the (a) EgoWalk-3 and (b) EgoWalk-4 Subsets Using Our CNN-Transformer Model. “Raw” Indicates That the Model Was Trained Using the Original Camera Poses. Subject-Specific Fine-Tuning Was Applied to the EgoWalk-4 Subset. The Best Results Are Marked in **Bold**

Method	Accuracy	Precision	Recall	F1

Raw	0.80	0.79	0.81	0.79
Pre-processed	**0.97**	**0.96**	**0.97**	**0.96**

(a) The EgoWalk-3 Subset				

Method	Accuracy	Precision	Recall	F1

Raw	0.69	0.77	0.75	0.73
Pre-processed	**0.95**	**0.96**	**0.96**	**0.96**

(b) The EgoWalk-4 Subset				

**TABLE VII:** Results of Using Camera Pose Sequences With Different Maximum Numbers of Frames for Subject-Specific Fine-Tuning on the EgoWalk-4 Subset. All Camera Pose Sequences Were Recorded at 30 Frames Per Second. The Best Results Are Marked in **Bold**

Max. # of Cam. Poses	Accuracy	Precision	Recall	F1

480 (default)	**0.95**	**0.96**	**0.96**	**0.96**
450	0.94	0.95	0.95	0.94
300	0.93	0.94	0.94	0.94
240	0.90	0.92	0.92	0.92
150	0.84	0.88	0.87	0.87

**TABLE VIII:** Performance Metrics (Accuracy, Precision, Recall, and F1-Score) for Different Walking Patterns on the EgoWalk-4 Subset

Metrics	Walking Pattem			
	Normal	M-L Instability	A-P Instability	Combined

Accuracy	1.00	0.97	1.00	0.86
Precision	1.00	0.96	0.91	0.96
Recall	1.00	0.97	1.00	0.86
F1	1.00	0.96	0.95	0.91

**TABLE IX:** Prediction Accuracy for Different Walking Patterns Across Three Subjects in the Test Set of the EgoWalk-4 Subset

Subject	Walking Pattern			
	Normal	M-L Instability	A-P Instability	Combined

Subject 1	1.00	1.00	1.00	0.98
Subject 2	1.00	1.00	1.00	0.67
Subject 3	1.00	0.92	1.00	0.89

## References

[1] Moreland B, Kakara R, and Henry A, “Trends in nonfatal falls and fall-related injuries among adults aged≥ 65 years-United States, 2012–2018,” Morbidity Mortality Weekly Rep., vol. 69, no. 27, pp. 875–881, Jul. 2020. 10.15585/mmwr.mm6927a5 PMC7732363 32644982

[2] Florence CS, Bergen G, Atherly A, Burns E, Stevens J, and Drake C, “Medical costs of fatal and nonfatal falls in older adults,” J. Amer. Geriatrics Soc., vol. 66, no. 4, pp. 693–698, 2018. 10.1111/jgs.15304 PMC6089380 29512120

[3] National Center for Injury Prevention and Control. (Sep. 4, 2023). Centers for Disease Control and Prevention Web-based Injury Statistics Query and Reporting System (wisqars). [Online]. Available: https://www.cdc.gov/falls/index.html

[4] Schoene D, Heller C, Aung YN, Sieber CC, Kemmler W, and Freiberger E, “A systematic review on the influence of fear of falling on quality of life in older people: Is there a role for falls?” Clin. Interventions Aging, vol. 14, pp. 701–719, Apr. 2019. 10.2147/CIA.S197857 PMC6514257 31190764

[5] Wang S, Liu X, and Pai Y-C, “Limb collapse or instability? Assessment on cause of falls,” Ann. Biomed. Eng., vol. 47, no. 3, pp. 767–777, Mar. 2019. 30617642 10.1007/s10439-018-02195-9 PMC6382554

[6] Pai Y-C and Patton J, “Center of mass velocity-position predictions for balance control,” J. Biomechanics, vol. 30, no. 4, pp. 347–354, Apr. 1997. 10.1016/s0021-9290(96)00165-0 9075002

[7] Pai Y-C, Wening JD, Runtz EF, Iqbal K, and Pavol MJ, “Role of feedforward control of movement stability in reducing slip-related balance loss and falls among older adults,” J. Neurophysiology, vol. 90, no. 2, pp. 755–762, Aug. 2003. 12904492 10.1152/jn.01118.2002

[8] Yang F, Anderson FC, and Pai Y-C, “Predicted threshold against backward balance loss following a slip in gait,” J. Biomechanics, vol. 41, no. 9, pp. 1823–1831, 2008. 10.1016/j.jbiomech.2008.04.005 PMC2515271 18538329

[9] Yang F, Espy D, and Pai Y-C, “Feasible stability region in the frontal plane during human gait,” Ann. Biomed. Eng., vol. 37, no. 12, pp. 2606–2614, Dec. 2009. 19760504 10.1007/s10439-009-9798-7 PMC2893223

[10] Kuptniratsaikul, “Effectiveness of simple balancing training program in elderly patients with history of frequent falls,” Clin. Interventions Aging, vol. 6, pp. 111–117, May 2011. 10.2147/CIA.S17851 PMC3095557 21594001

[11] Lord SR, Menz HB, and Tiedemann A, “A physiological profile approach to falls risk assessment and prevention,” Phys. Therapy, vol. 83, no. 3, pp. 237–252, Mar. 2003. 12620088

[12] Delbaere K, Close JCT, Mikolaizak AS, Sachdev PS, Brodaty H, and Lord SR, “The falls efficacy scale international (FES-I). A comprehensive longitudinal validation study,” Age Ageing, vol. 39, no. 2, pp. 210–216, Mar. 2010. 20061508 10.1093/ageing/afp225

[13] Pai Y-C, Bhatt T, Yang F, and Wang E, “Perturbation training can reduce community-dwelling older Adults’ annual fall risk: A randomized controlled trial,” J. Gerontol. A, Biol. Sci. Med. Sci., vol. 69, no. 12, pp. 1586–1594, Dec. 2014. 24966227 10.1093/gerona/glu087 PMC4296119

[14] Lu N, Wu Y, Feng L, and Song J, “Deep learning for fall detection: Three-dimensional CNN combined with LSTM on video kinematic data,” IEEE J. Biomed. Health Informat., vol. 23, no. 1, pp. 314–323, Jan. 2019. 10.1109/JBHI.2018.2808281 29994460

[15] Salimi M, Machado JJM, and Tavares JMRS, “Using deep neural networks for human fall detection based on pose estimation,” Sensors, vol. 22, no. 12, p. 4544, Jun. 2022. 35746325 10.3390/s22124544 PMC9229309

[16] Wu L , “Video-based fall detection using human pose and constrained generative adversarial network,” IEEE Trans. Circuits Syst. Video Technol., vol. 34, no. 4, pp. 2179–2194, Apr. 2024.

[17] Qi P, Chiaro D, and Piccialli F, “FL-FD: Federated learning-based fall detection with multimodal data fusion,” Inf. Fusion, vol. 99, Nov. 2023, Art. no. 101890.

[18] Rana SP, Dey M, Ghavami M, and Dudley S, “Markerless gait classification employing 3D IR-UWB physiological motion sensing,” IEEE Sensors J., vol. 22, no. 7, pp. 6931–6941, Apr. 2022.

[19] Burduk R, Rojek I, Mikołajewska E, and Mikołajewski D, “Post-stroke gait classification based on feature space transformation and data labeling,” Appl. Sci., vol. 12, no. 22, p. 11346, Nov. 2022.

[20] Wang X, Ellul J, and Azzopardi G, “Elderly fall detection systems: A literature survey,” Frontiers Robot. AI, vol. 7, p. 71, Jun. 2020. 10.3389/frobt.2020.00071 PMC7805655 33501238

[21] Xu T, Zhou Y, and Zhu J, “New advances and challenges of fall detection systems: A survey,” Appl. Sci., vol. 8, no. 3, p. 418, Mar. 2018.

[22] Khan SS and Hoey J, “Review of fall detection techniques: A data availability perspective,” Med. Eng. Phys., vol. 39, pp. 12–22, Jan. 2017. 27889391 10.1016/j.medengphy.2016.10.014

[23] Ren L and Peng Y, “Research of fall detection and fall prevention technologies: A systematic review,” IEEE Access, vol. 7, pp. 77702–77722, 2019.

[24] Imamura T, Moshnyaga VG, and Hashimoto K, “Automatic fall detection by using Doppler-radar and LSTM-based recurrent neural network,” in Proc. IEEE 4th Global Conf. Life Sci. Technol. (LifeTech), Mar. 2022, pp. 36–37.

[25] Ma L, Li X, Liu G, and Cai Y, “Fall direction detection in motion state based on the FMCW radar,” Sensors, vol. 23, no. 11, p. 5031, May 2023. 37299758 10.3390/s23115031 PMC10255840

[26] Sarshar M, Polturi S, and Schega L, “Gait phase estimation by using LSTM in IMU-based gait analysis—Proof of concept,” Sensors, vol. 21, no. 17, p. 5749, Aug. 2021. 34502640 10.3390/s21175749 PMC8433817

[27] Liu K, Liu Y, Ji S, Gao C, Zhang S, and Fu J, “A novel gait phase recognition method based on DPF-LSTM-CNN using wearable inertial sensors,” Sensors, vol. 23, no. 13, p. 5905, Jun. 2023. 37447755 10.3390/s23135905 PMC10347001

[28] Sadeghzadehyazdi N, Batabyal T, and Acton ST, “Modeling spatiotemporal patterns of gait anomaly with a CNN-LSTM deep neural network,” Expert Syst. Appl., vol. 185, Dec. 2021, Art. no. 115582.

[29] Chen B , “Computer vision and machine learning-based gait pattern recognition for flat fall prediction,” Sensors, vol. 22, no. 20, p. 7960, Oct. 2022. 36298311 10.3390/s22207960 PMC9612353

[30] Setiawan F, Liu A-B, and Lin C-W, “Development of neurodegenerative Diseases’ gait classification algorithm using convolutional neural network and wavelet coherence spectrogram of gait synchronization,” IEEE Access, vol. 10, pp. 38137–38153, 2022.

[31] Wang Q, Zeng W, and Dai X, “Gait classification for early detection and severity rating of Parkinson’s disease based on hybrid signal processing and machine learning methods,” Cognit. Neurodynamics, vol. 18, no. 1, pp. 109–132, Feb. 2024. 10.1007/s11571-022-09925-9 PMC10881932 38406205

[32] Li W , “Real-time fall detection using mmWave radar,” in Proc. IEEE Int. Conf. Acoust., Speech Signal Process. (ICASSP), May 2022, pp. 16–20.

[33] Shi L-F, Liu Z-Y, Zhou K-J, Shi Y, and Jing X, “Novel deep learning network for gait recognition using multimodal inertial sensors,” Sensors, vol. 23, no. 2, p. 849, Jan. 2023. 36679646 10.3390/s23020849 PMC9867501

[34] Wang C-Y, Sadrieh FK, Shen Y-T, Oppizzi G, Zhang L-Q, and Tao Y, “Real-time privacy-preserving fall risk assessment with a single body-worn tracking camera,” in Proc. ICASSP - IEEE Int. Conf. Acoust., Speech Signal Process. (ICASSP), Apr. 2024, pp. 1866–1870.

[35] Charfi I, Miteran J, Dubois J, Atri M, and Tourki R, “Optimised spatio-temporal descriptors for real-time fall detection: Comparison of SVM and AdaBoost based classification,” J. Electron. Imag., vol. 22, no. 14, p. 17, 2013.

[36] Adhikari K, Bouchachia H, and Nait-Charif H, “Activity recognition for indoor fall detection using convolutional neural network,” in Proc. 15th IAPR Int. Conf. Mach. Vis. Appl. (MVA), May 2017, pp. 81–84.

[37] Charfi I, Miteran J, Dubois J, Atri M, and Tourki R, “Definition and performance evaluation of a robust SVM based fall detection solution,” in Proc. 8th Int. Conf. Signal Image Technol. Internet Based Syst., Nov. 2012, pp. 218–224.

[38] Ma X, Wang H, Xue B, Zhou M, Ji B, and Li Y, “Depth-based human fall detection via shape features and improved extreme learning machine,” IEEE J. Biomed. Health Informat., vol. 18, no. 6, pp. 1915–1922, Nov. 2014. 10.1109/JBHI.2014.2304357 25375688

[39] Zhang Z, Conly C, and Athitsos V, “Evaluating depth-based computer vision methods for fall detection under occlusions,” in Proc. 10th Int. Symp. Adv. Vis. Computing (ISVC), Dec. 2014, pp. 196–207.

[40] Saleh M, Abbas M, and Le Jeannès RB, “FallAllD: An open dataset of human falls and activities of daily living for classical and deep learning applications,” IEEE Sensors J., vol. 21, no. 2, pp. 1849–1858, Jan. 2021.

[41] Sucerquia A, López JD, and Vargas-Bonilla JF, “SisFall: A fall and movement dataset,” Sensors, vol. 17, no. 1, p. 198, Jan. 2017. 28117691 10.3390/s17010198 PMC5298771

[42] Yu X, Jang J, and Xiong S, “A large-scale open motion dataset (KFall) and benchmark algorithms for detecting pre-impact fall of the elderly using wearable inertial sensors,” Frontiers Aging Neurosci., vol. 13, Jul. 2021, Art. no. 692865. 10.3389/fnagi.2021.692865 PMC8322729 34335231

[43] Frank K, Vera Nadales MJ, Robertson P, and Pfeifer T, “Bayesian recognition of motion related activities with inertial sensors,” in Proc. 12th ACM Int. Conf. Adjunct Papers Ubiquitous Comput. Adjunct, Sep. 2010, pp. 445–446.

[44] Vavoulas G, Pediaditis M, Chatzaki C, Spanakis EG, and Tsiknakis M, “The MobiFall dataset: Fall detection and classification with a smartphone,” in Proc. Artif. Intell., Concepts, Methodologies, Tools, Appl., Jul. 2017, pp. 1218–1231.

[45] Casilari E, Santoyo-Ramón JA, and Cano-García JM, “UMAFALL: A multisensor dataset for the research on automatic fall detection,” Proc. Comput. Sci., vol. 110, pp. 32–39, Jan. 2017.

[46] Kwolek B and Kepski M, “Human fall detection on embedded platform using depth maps and wireless accelerometer,” Comput. Methods Programs Biomed., vol. 117, no. 3, pp. 489–501, Dec. 2014. 25308505 10.1016/j.cmpb.2014.09.005

[47] Martínez-Villaseñor L, Ponce H, Brieva J, Moya-Albor E, Núñez-Martínez J, and Peñafort-Asturiano C, “UP-fall detection dataset: A multimodal approach,” Sensors, vol. 19, no. 9, p. 1988, Apr. 2019. 31035377 10.3390/s19091988 PMC6539235

[48] Nguyen T-N, Huynh H-H, and Meunier J, “Skeleton-based abnormal gait detection,” Sensors, vol. 16, no. 11, p. 1792, Oct. 2016. 27792181 10.3390/s16111792 PMC5134451

[49] Khokhlova M, Migniot C, Morozov A, Sushkova O, and Dipanda A, “Normal and pathological gait classification LSTM model,” Artif. Intell. Med., vol. 94, pp. 54–66, Mar. 2019. 30871683 10.1016/j.artmed.2018.12.007

[50] Jun K, Lee Y, Lee S, Lee D-W, and Kim MS, “Pathological gait classification using Kinect v2 and gated recurrent neural networks,” IEEE Access, vol. 8, pp. 139881–139891, 2020.

[51] Goldberger AL , “PhysioBank, PhysioToolkit, and PhysioNet: Components of a new research resource for complex physiologic signals,” Circulation, vol. 101, no. 23, pp. e215–e220, Jun. 2000. 10851218 10.1161/01.cir.101.23.e215

[52] Grunnet-Jepsen A, Harville M, Fulkerson B, Piro D, Brook S, and Radford J, “Introduction to Intel realsense visual SLAM and the t265 tracking camera. Product documentation,” Intel company, 2019.

[53] Vaswani A , “Attention is all you need,” in Proc. Adv. Neural Inf. Process. Syst., vol. 30, Jun. 2017, pp. 5998–6008.

[54] Maaten L. v. d. and Hinton GE, “Visualizing data using t-SNE,” J. Mach. Learn. Res., vol. 9, no. 86, pp. 2579–2605, Jan. 2008.

[55] Dong Y, Yan D, Li T, Xia M, and Shi C, “Pedestrian gait information aided visual inertial SLAM for indoor positioning using handheld smartphones,” IEEE Sensors J., vol. 22, no. 20, pp. 19845–19857, Oct. 2022.

[56] Ren P, Elyasi F, and Manduchi R, “Smartphone-based inertial odometry for blind walkers,” Sensors, vol. 21, no. 12, p. 4033, Jun. 2021. 34208112 10.3390/s21124033 PMC8230905

[57] Jin L and Ye C, “Visual-LiDAR-Inertial odometry: A new visual-inertial SLAM method based on an iPhone 12 pro,” in Proc. IEEE/RSJ Int. Conf. Intell. Robots Syst. (IROS), Oct. 2023, pp. 1511–1516.

[58] Nguyen TN, Huynh HH and Meunier J, “3D reconstruction with time-of-flight depth camera and multiple mirrors,” IEEE Access, vol. 6, pp. 38106–38114, 2018. [Online]. Available: https://www-labs.iro.umontreal.ca/labimage/GaitDataset/

[59] Chen C, Lu X, Markham A, and Trigoni N, “IONet: Learning to cure the curse of drift in inertial odometry,” in Proc. AAAI Conf. Artif. Intell., vol. 32, Feb. 2018, pp. 6468–6476.

[60] Herath S, Yan H, and Furukawa Y, “RoNIN: Robust neural inertial navigation in the wild: Benchmark, evaluations, & new methods,” in Proc. IEEE Int. Conf. Robot. Autom. (ICRA), May 2020, pp. 3146–3152.

